# Nasogastric tube feeding under restraint: understanding the impact and improving care

**DOI:** 10.1192/bjb.2023.58

**Published:** 2024-06

**Authors:** Sarah J. Fuller, Jacinta Tan, Dasha Nicholls

**Affiliations:** 1 Imperial College London, UK; 2 Northamptonshire Healthcare NHS Foundation Trust, UK; 3 Oxford Health NHS Foundation Trust, UK; 4 University of Oxford, UK

**Keywords:** Nasogastric feeding, physical restraint, compulsory treatment, restrictive practices, eating disorders

## Abstract

**Background:**

Nasogastric tube feeding under physical restraint is an intervention that clinicians working in specialist mental health in-patient units may need to implement.

**Aims:**

To examine the impact of this intervention on people with lived experience, carers and staff.

**Method:**

People with lived experience and parents and/or carers were recruited via UK eating disorder charity Beat. Clinicians were recruited via the British Eating Disorders Society's research forum. Qualitative semi-structured interviews were conducted and transcribed, and the results were thematically analysed.

**Results:**

Thirty-six participants took part, and overlapping themes were identified. All participants spoke in relation to four themes: (a) the short-term impact on the patient; (b) the impact on those around the patient; (c) the long-term impact; and (d) the positive impact. Subthemes were identified and explored.

**Conclusion:**

This lifesaving intervention can also negatively affect patients, parents and carers, peers and staff. Further research is needed to understand how interactions and environmental modifications can mitigate the negative impacts.

Nasogastric tube (NGT) feeding under physical restraint is a highly coercive and restrictive intervention that can be used to save the lives of patients who are extremely medically compromised secondary to a psychiatric condition, such as patients with restrictive eating disorders. Anorexia nervosa is an ego-syntonic illness, and patients may value their illness to the extent that their recovery is hindered.^1^ As such, restrictive interventions can be welcomed and may even reinforce the anorexic identity.^2^ Reducing the use of restrictive interventions requires a thorough understanding of the factors that contribute to their instigation, the decision-making processes involved and their impact, in order to identify possible alternative strategies.

There is some evidence regarding the impact of NGT feeding in eating disorders where the patient consents. NGT feeding is a safe way to help patients restore physical health, given the high nutritional demands of refeeding in the context of malnutrition.^3^ However, there has been little research into the impact of this intervention when delivered against the will of the patient under physical restraint. One previous study investigated the impact on nursing assistants and identified three themes: first, delivering the intervention was described as an unpleasant experience, whereby staff reported being stressed, exhausted and injured; second, the importance of coping by talking to colleagues and young people in recovery was identified; and, finally, nursing assistants reported becoming desensitised to delivering this intervention.^4^ Recently published research highlights the extent of this intervention in mental health units in England, with 622 patients being reported as requiring this intevention in a one-year period, and the time this intervention required varying from a one-off NGT feed under physical restraint to 312 weeks (mean = 21.9 weeks).^5^ In one of a series of papers exploring NGT feeding under restraint in mental health wards, we aimed to investigate the impact of this intervention on people with lived experience of NGT feeding under restraint and their parents and/or carers, as well as the impact on the wider multidisciplinary team (MDT).^6^

## Method

### Design

The methodology for this study was co-created with a project steering group comprising people with lived experience (described hereafter as patients) and parent and/or carer representatives, clinicians and academics. As advised by the steering group, patients were interviewed individually because of the personal and confidential nature of their narratives, whereas parent and/or carer experts by experience were interviewed in groups so that participants could benefit from peer support during the interview process. We also interviewed clinicians from both adult's and children and young people's units, across a range of in-patient MDT members that facilitated this intervention.

Across the three participant groups, each participant had an interview that lasted up to 90 min and was based on a semi-structured topic guide. The topic guides took the following format. (a) Introductory questions to set the participant at ease and ask whether they had any questions (5–10 min). (b) What was your experience of NGT feeding under physical restraint? (20 min). (c) What is the impact of NGT feeding under physical restraint? Is it supportive or are there harms from this intervention? (20 min). (d) Why do you think some people require physical restraint for NGT feeding? (10 min). (e) In their opinion, what helps patients no longer need NGT feeding under physical restraint (20 min). (f) Is there anything else relating to this topic that you would like to talk about? (10 min).

### Inclusion and exclusion criteria

Participants who were previously patients had received NGT feeding under restraint during their treatment, were at least 1 year post-discharge from mental health in-patient care and were not involved in litigation regarding their treatment. Participants who were parents and/or carers were those who had a loved one who had received this intervention in an in-patient mental health setting (excluding those who had experienced the intervention only in acute medical settings). Clinicians were healthcare professionals working in an in-patient mental health setting where this intervention was carried out and had been part of the MDT where clinical discussions were held.

### Procedure

Patients and parents and/or carers were recruited via online advertising from Beat, the UK eating disorders charity, and clinicians were recruited by advertising through the British Eating Disorders Society. Potential participants were e-mailed the participant information sheet and consent form to sign and return to the researcher. An interview was then conducted remotely via Microsoft Teams videoconferencing software.

The semi-structured topic guide was co-produced by the steering group, and audio of the interviews was recorded and transcribed using Teams software. Participants were asked whether they had questions regarding the research or their participation upon registering their interest to participate and were asked to reconfirm their consent verbally at the time of interview.

Imperial College London's Research Ethics Committee granted ethical approval (reference number 21IC7157). As participants were not recruited via the National Health Service (NHS), ethical approval was not sought via the Health Research Authority.

### Data analysis

This research project had a qualitative design and used thematic analysis based on principles outlined by Braun and Clarke.^5^ Six phases were used to explore patterns and identify themes: (a) initial familiarisation was achieved by reading the transcripts multiple times; (b) a coding frame was developed by manual line-by-line exploration of the data; (c) the coding frame was validated with J.T. and D.N. using specific examples; (d) transcripts were coded; (e) triangulation between participant groups was performed; (f) validation was conducted with research leads and the study steering group.

### Consent statement

Participants were provided with a study information sheet and completed an electronic consent form.

## Results

### Participants

There were 36 participants across patients (*n* = 7), parents and/or carers (*n* = 13) and clinicians (*n* = 16). As advised by the steering group, seven individual interviews were conducted with past patients who had received the intervention, as well as three group interviews with parents or carers (3–6 per group) and 16 individual interviews with clinicians.

### Patient participants

Seven female participants were recruited; all had been diagnosed with anorexia nervosa and had experienced NGT feeding under physical restraint during mental health in-patient treatment. At the time of interview, ages ranged from 19–54 years. The reported duration of illness ranged from 3 to over 30 years. The number of admissions per patient ranged from one to 13; the shortest admission was reported as 8 months and the longest was 5 years. Some participants were recalling recent experiences (14 months ago), whereas one participant reported experiences from three decades ago. Participants recounted admissions from NHS and independent sector units. Child and adolescent mental health (CAMH) admissions were in different settings, including general adolescent units (GAU), psychiatric intensive care units (PICU), low secure units (LSU) and CAMH specialist eating disorder units (SEDU), whereas the adult admissions were predominantly to SEDU.

### Parents/carers

Ten mothers, one step-mother and two fathers participated across three group interviews; all represented daughters that had been NGT fed under physical restraint. Their daughters were aged 12–27 years old at the time of interview. One parent reported their daughter receiving this intervention twice, whereas another parent reported a 7-year history of back-to-back in-patient admissions during which this intervention was required multiple times over many months. These parents represented admissions to CAMH GAU, PICU and SEDU and to adult SEDU only. None were related to the patient participants.

### Staff

Of the sixteen staff interviewed, five were male and 11 were female. Clinical experience working with patients with eating disorders in mental health settings ranged from 10 months to 17 years. Staff were from seven professional backgrounds (psychiatry, psychology, dietetics, occupational therapy, nursing, healthcare assistants and peer support workers). Participants had experience working in services that spanned NHS and private units, in CAMH GAU, SEDU, LSU, medium secure units and PICU, and adult SEDU and LSU.

#### Thematic analysis

There was significant overlap in the generated themes and associated subthemes. A powerful narrative across all three groups was of the complex and emotionally fraught impact of the intervention on the patients, parents and staff. The results are presented accordingly in Table 1.

**Table 1:** Summary of all the themes identified across participant groups

Impact of NGT feeding under restraint	Impact on the patient during the admission	Trauma
		Strengthening of illness
		Breakdown of trust
		Impact of compulsory treatment
		Impact on others
		Isolation and identity
	Impact on others of patients being fed via NGT under restraint	On peers
		On carers/parents
		On staff
	Longer-term impact on the patient who experienced NGT feeding under restraint	Flashbacks
		PTSD
	Positive impact of NGT feeding under restraint	On the patient
		On parents/carers
		On staff

NGT, nasogastric tube; PTSD, post-traumatic stress disorder.

#### Impact of NGT feeding under restraint – on the patient

Participants who had experienced NGT feeding under restraint spoke about this in terms of the impact of the intervention on themselves and how it changed their relationship with the other patients around them.

*Participant 16, patient: I think it was eight staff members … they just restrained me and tubed me … I remember screaming at them like ‘don't like don't do this’ and I was absolutely petrified ‘cause just for me it was more like not necessarily calories at the time, it was just the [pause] intrusiveness of having an NG shoved down me … then later when you go back to the group [of other patients] they hate you because of it too*.

Many staff members were aware of the damage this intervention can do to patients requiring it:

*Participant 28, clinician: It is a breakdown of trust. It destroys the therapeutic relationship … Uhm, it is non-therapeutic in the psychological context. So I think … there will always be more reasons not to do it than to do it*.

There were other more negative and inadvertent consequences of NGT feeding under restraint. For some participants, it set a new bar for treatment that ‘had’ to be achieved and was difficult to move on from.

*Participant 18, patient: Once you have crossed over into that new form of treatment … there's a danger people are going to become wedded to that and feel it's really hard to come off the [NG] tube. They've been on the tube once, they will feel like they need to go back on it. It's just too tantalising … I'm sectioned, on the tube, being restrained. Like it just starts to set up a benchmark*.

Simultaneously, participants reflected on how this treatment was bound up with wider issues – such as the isolation they felt during their admission, the strength of identification and alliance they developed with their eating disorder, and its exacerbation by alienation from fellow in-patients in a way that was potentially reinforcing of the illness. All these were inadvertent consequences of the coercive experience of NGT feeding under restraint.

*Participant 16, patient: You can just live in, like, a bubble, and the only thing that's important is that meal, snack or feed. And yeah, you lose track of all reality. It's another step away from the real world, you are even isolated on the unit as the other patients don't like to see the tube, let alone hear your restraint* *…* *I felt as if even the other patients didn't understand me, nobody did except my anorexia*.

#### Impact of NGT feeding under restraint – on others

Patient participants reported the negative impact on themselves of hearing or seeing other patients being restrained for their NGT feeds, along with a parent/carer reflection on how this was unhelpful to all the patients.

*Participant 19, patient: I did find that very, very difficult, when she first had the tube because it took me right back [to when I experienced it myself] and I struggled. […] Her screams still haunt me but I think it did impact some of the other girls as well* *…* *even though some of them had never had tubes*.

*Participant 4, parent/carer: There was something about the layout of the building, which was actually really not helpful because the patients could hear other patients screaming and shouting. So its super triggering – no wonder they struggle to eat with all that going on all day*.

A number of parents described having been traumatised by hearing screaming on visits to the units:

*Participant 4, parent/carer: So, we would be downstairs in the waiting area, and we could hear our own kid being restrained [for a feed]. You know your own child's scream. That was so, so unbelievably traumatising*.

Across the parent group interviews, parents reflected on how difficult delivering this intervention was for the staff group of the wards:

*Participant 7, parent/carer: You could see there were days when the staff were just worn down after doing one feed after another. I have seen staff with tears in their eyes after difficult restraints*.

#### Longer-term post-traumatic impact of NGT feeding under restraint

Patient and parent participants spoke of longer-term post-traumatic impact, such as subsequently seeing or carrying out the intervention on others, having previously experienced it themselves, or the impact of having healthcare professionals go near their nose in contexts other than NGT feeding**.**

*Participant 14, patient [who works on a paediatric unit]: Uhm, I will like pass NGTs now, not normally on big kids, on babies or whatever* *…* *but I can actually feel it going down my throat, like* *…* *and, like, you know the, like, jelly or whatever they used to use. I can smell it. I can taste it sometimes*.

*Participant 4, parent/carer; My daughter was really triggered by the [COVID-19] PCR tests* *…* *you know they stick something up your nose and when she first went to have to have that she said that produced a huge flashback. In fact she now refuses them* *…* *My daughter is so traumatised after being NG fed like that she's been diagnosed with PTSD [post-traumatic stress disorder] and even had some EMDR, but actually that was too much for her* *…* *she might revisit that at some point, but she has flashbacks*.

#### The positive impact of NGT feeding under restraint

A theme emerged of being grateful for the NGT feeding under restraint as part of a difficult but necessary journey to recovery from the eating disorder. This was spoken about within all three groups.

*Participant 18, patient: The reality is, was, traumatising absolutely 100%, but it saved my life. So how do I square that one, like, it's quite a confusing legacy of care to be left with. I'm OK with that, it has allowed me a life, a job, a relationship, a future*.

*Participant 3, parent/carer: I mean she is alive, she has a chance at living again after this terrible, terrible, illness so I am grateful for that*.

*Participant 21, clinician: that moment when they go home and you know they will do well, you reflect on just how ill they were, how hard you had to fight to get them better. Sometimes you bump into them, like, elsewhere, and you get a massive hug, that's the best feeling*.

## Discussion

This is the first study to explore the views of multiple stakeholders – patients, their parents/carers and clinicians – regarding their experience of NGT feeding under restraint. Across these groups there was acknowledgment that this can be a lifesaving intervention, with the short-term benefit of medical stabilisation and the long-term reflection that this can be a turning point in someone's treatment journey that allows them to fully recover. This aligns with previous research^7^ where participants, once recovered, reported gratitude for receiving compulsory intervention. However, it is clear from the accounts of participants in this study that NGT feeding under restraint is traumatising to all involved.

A strong narrative of the negative psychological impact of being NGT fed under restraint was identified by all participants. This is mirrored in other qualitative research where patients with anorexia nervosa spoke about their experience of being detained under the Mental Health Act for treatment^13^ – how this led to ‘rebellion’ and ‘digging their heels in’, and how ‘services responded with increasingly restrictive interventions (i.e. tube feeding …)’.^2^ However, ours is the first study to find that this intervention can lead to recipients being diagnosed with PTSD, suggesting that potential for long-term harm needs to be balanced against the short- and long-term benefits of this clinical intervention. The ethical and clinical justifications for how and when NGT feeding under restraint is used are critical to this equation. In cases where NGT feeding under restraint is used repeatedly over prolonged periods,^6^ there are legitimate grounds for concern, underlined by our findings. Starting NGT feeding under restraint may result in inadvertent worsening of the dynamics, reinforcing the refusal of nutrition^2^ and leaving no clear ‘exit strategy’ to discontinue the intervention and allow the patient to transition to voluntary NGT feeding or oral nutrition. It is particularly concerning if the practice of NGT feeding under restraint is prolonged, with continued suffering and loss of liberty involved. Prolonged use of non-consented treatment has the potential to affect the integrity of the sense of self, personal dignity and the sense of being in control of oneself.^8,9^

### Strengths and limitations

There has been very little research into NGT feeding under physical restraint, and this study adds to the sparse existing knowledge by exploring the impact of the intervention on patients, their peers and families, and the staff looking after them. The limitations to this research include that the views were restricted to patients with a diagnosis of anorexia nervosa and may not be generalisable to those with other diagnoses likely to receive the intervention; that the age and stage of illness of the patients at the time of the intervention was not examined as a factor in how the intervention was experienced; and that views were sought only from those within England and therefore may not represent experiences in other countries, particularly where legislation and practices may differ. Furthermore, the seven experts by experience represented the smallest group within the sample, and the themes for this group may not have reached saturation.

### Future research

NGT feeding under restraint is a highly contentious area, requiring more research in terms of understanding the ethical issues, clinical efficacy, and short- and long-term impact. We suggest that the findings of our study on the impact of the experience on patients, carers and staff justify consideration of and further research into how clinical practice should be modified to minimise harm, while simultaneously recognising the lifesaving nature of the intervention. For example, the impact of distress and screaming could be mitigated by measures such as soundproof treatment rooms or offering ear defenders or similar to others on the unit when the intervention is needed. Furthermore, our previous research^10^ has highlighted that the decision-making process regarding this intervention is complex, with potential for iatrogenic harm. A possible future research direction would be to identify whether taking decisions regarding the need for NGT feeding under physical restraint in collaboration with the patient in the context of advance care planning reduces the traumatic impact.

### Clinical implications

This study highlights how NGT feeding under restraint is considered by patients, carers and clinicians as sometimes necessary but highly restrictive and not without negative effects. These include not just effects on the patients who receive the intervention but also the impact on their families, the clinicians delivering the procedure and other patients in the unit. There are short-term consequences, which can include inadvertent entrenchment of the eating disorder, isolation within the unit and increased conflict; and also long-term consequences such as PTSD and other post-traumatic effects.

Existing guidance^11,12^ shows how dietetic practice can be adapted to reduce the time a person spends in physical restraint for NGT feeding. Units should be aware of this guidance and adopt it when physical restraint is needed, to reduce the unwanted impact of feeding on other patients, their families and staff. This guidance should be extended to include strategies that reduce the frequency and duration of NGT feeding under restraint.

## About the authors

**Sarah J. Fuller** is the Clinical Lead Dietitian for Northamptonshire Healthcare NHS Foundation Trust and Research Dietitian at Imperial College London, UK. **Jacinta Tan** is a Child and Adolescent Psychiatrist at Oxford Health NHS Foundation Trust, Oxford, UK and at the University of Oxford, UK. **Dasha Nicholls** is a Reader in Child Psychiatry in the Division of Psychiatry, Imperial College London, UK.

## Data Availability

The data that support the findings of this study are available from the corresponding author, S.J.F., upon reasonable request.
